# Applying Peplau's Interpersonal Relations Theory to Enhance Older Adults’ Health Literacy Through Nursing Care: A Theory Analysis

**DOI:** 10.1177/08980101251345027

**Published:** 2025-06-17

**Authors:** Steven Hall, Kathleen Shearer

**Affiliations:** 3158 University of Alberta, Edmonton, Canada

**Keywords:** nursing theory, Hildegard Peplau, interpersonal relations, health literacy, older adults

## Abstract

This paper explores the utility of Peplau's Interpersonal Relations Theory in enhancing health literacy among older adults in clinical nursing practice. With an aging global population, older adults increasingly require complex healthcare interactions. Effective communication between nurses and older adults, a central tenet of Peplau's theory, is critical for promoting health literacy and ensuring positive outcomes. Health literacy enables older adults to better navigate healthcare systems, manage chronic conditions, and engage in self-care. This theory analysis employs Walker and Avant's methodology to examine Peplau's theory in the context of enhancing older adults’ health literacy. We provide a historical overview of the theory's origins, describe its meaning, and present a literature review of academic literature that explores the concept of nurses enhancing older adults’ health literacy. Through a thematic synthesis of nine records retrieved from three databases (MEDLINE, CINAHL, PsycINFO), this study identifies key themes such as trust-building, the role of social support, and nurses’ roles as educators in facilitating health literacy. Findings support interpersonal nurse-patient relationships as critical to addressing health literacy barriers. The analysis highlights that Peplau's Interpersonal Relations Theory provides a useful framework for improving health literacy among older adults, ultimately supporting their well-being and healthcare outcomes.

## Background

Population aging is a global phenomenon, leading to a significant shift in demographics (World Health Organization, 2020). Older adults often have multiple chronic conditions, and their interaction with healthcare systems becomes more frequent and complex (Parsons et al., 2021; Tinetti et al., 2019). However, barriers such as socioeconomic status, geographic location, and system inefficiencies can limit this access (Baciu et al., 2017; McMaughan et al., 2020). Older adults in rural areas, for example, face challenges in healthcare access, which is associated with higher rates of disability and cognitive impairment (McMaughan et al., 2020; Parsons et al., 2021). Older adults’ interaction with healthcare professionals, particularly nurses, is essential in managing their complex healthcare needs and promoting overall well-being (Fathidokht et al., 2023; Jack et al., 2024). Effective communication is a central tenet in these interactions, described as an exchange of information, thoughts, and feelings to successfully produce a desired or intended result, which is usually satisfaction in care (Afriyie, 2020).

Compared with other healthcare professionals, nurses also often spend the most time with patients, developing significant nurse-patient relationships (Kwame & Petrucka, 2021). Nurses often serve as primary points of contact, providing care and support (Fathidokht et al., 2023). As such, nurses’ ability to communicate health information effectively can directly influence older adults’ healthcare outcomes (Jack et al., 2024; Karl & Mion, 2020). With nurses continually being ranked the most trusted profession (National Nurses United, 2024), relationship building is necessary to mitigate the prevalence of health misinformation in today's age (Suarez-Lledo & Alvarez-Galvez, 2021). The purpose of this paper is to describe the utility of Peplau's Interpersonal Relations Theory in the clinical practice context of nurses enhancing older adults’ health literacy and to support these linkages through a review and evidence synthesis of literature.

### Peplau's Interpersonal Relations Theory

Peplau's Interpersonal Relations Theory, theorized by Hildegard Peplau in the 1950s, is one of the most influential theories in nursing (Hagerty et al., 2017). Peplau's theory emphasizes the significance of the nurse-patient relationship, suggesting that nursing is fundamentally an interpersonal, therapeutic process (Peplau, 1992, 1997). The central idea of this theory is that communication and interactions between nurses and patients serve to promote patients’ well-being and facilitate their recovery (Hagerty et al., 2017). While several frameworks exist that emphasize communication and patient education principles (e.g., Health Belief Model or Social Cognitive Theory), Peplau's theory was intentionally selected for this analysis due to its grounding in nursing practice and its focus on the therapeutic nurse-patient relationship – an aspect highly relevant to older adults’ health literacy.

Although developed in the 1950s, we argue that Peplau's Interpersonal Relations Theory remains applicable in the context of enhancing the health literacy of older adults. Its enduring relevance offers a theoretical lens rooted in therapeutic communication that complements contemporary models rather than competes with them. In today's climate of health misinformation and systemic barriers to care, applying a relationship-centered nursing theory offers both practical relevance and a holistic, personalized approach to improving health literacy outcomes among older adults. Demonstrating the utility of Peplau's theory in this context revisits its importance in present-day settings where nursing care is delivered.

### Connection to Holistic Nursing

Holistic nursing focuses on addressing the interconnected physical, emotional, social, and cognitive dimensions of care (Frisch & Rabinowitsch, 2019), which aligns with Peplau's emphasis on the therapeutic nurse-patient relationship. Peplau's theory's structure, explored within this manuscript, reflects the principles of holistic nursing by fostering trust, promoting mutual understanding, and empowering patients through personalized care and communication strategies. Integrating Peplau's theory into nursing practice with older adults supports the holistic nursing goal of creating a supportive, patient-centered environment that enhances older adults’ ability to navigate healthcare systems, manage chronic conditions, and achieve improved overall well-being.

### Health Literacy

Health literacy is vital in empowering older adults to understand and make informed decisions regarding their healthcare (Eronen et al., 2021; Liu et al., 2020). Adequate health literacy enables them to better navigate the healthcare system, comprehend medical information, adhere to treatment plans, and engage in self-care and self-management practices (Liu et al., 2020; Palumbo et al., 2021). A recent concept analysis (Parnell et al., 2019) examined health literacy, specifically in the context of nursing practice. Nurses must be aware of the system-related challenges patients face and strive to enhance their patients’ health literacy during interpersonal relations through targeted education and communication strategies (Parnell et al., 2019). Parnell et al. (2019) argue that health literacy is a dynamic and quickly changing concept. Therefore, defining the utility of a timeless nursing theory in this context is worthwhile. As well, communication is the underlying process by which health literacy is built (Nantsupawat et al., 2020), so a theory that informs this process is beneficial to explore. Furthermore, considering that older adults are one of the more frequent populations of healthcare users (Parsons et al., 2021; Tinetti et al., 2019), health literacy is a critical factor in influencing the health outcomes of older adults. Peplau's theory is one of the few foundational nursing theories that explicitly positions the nurse in promoting patient well-being through interpersonal communication and relationship-building, which are central to health literacy development. As Parnell et al. (2019) note: “Health literacy depends on reciprocal communication between the individual and provider. Therefore, both the individuals’ and the providers’ social and interpersonal skills affect the level of health literacy” (p. 322).

## Methods

A theory analysis aids healthcare professionals in understanding and incorporating theoretical knowledge into everyday practice (Elliott et al., 2002; Walker & Avant, 2019). This process can identify challenges, benefits, and practical limitations of using theory in real-world settings (Elliott et al., 2002). We chose the method of theory analysis to use Peplau's Interpersonal Relations Theory as a lens to explore literature of nurses working to enhance older adults’ health literacy, to demonstrate its utility and applicability within clinical practice reports in academic literature, and to bring attention once again to this foundational nursing theory.

This theory analysis employed the six-step methodology defined by Walker and Avant (2019). The six steps to theory analysis involve: (1) identifying the origins of the theory, (2) examining the meaning of the theory, (3) analyzing the logical adequacy of the theory, (4) determining the usefulness of the theory, (5) defining the degree of generalizability and the parsimony of the theory, and (6) determining the testability of the theory (Walker & Avant, 2019). Each of these steps is described in detail herein:

### 1) Identify the Origins of the Theory

We reviewed literature about Peplau's Interpersonal Theory. Journal articles, as well as three print books (Forchuk, 1993; Peplau, 1991; Simpson & Peplau, 1991) that discussed the theory's origins were retrieved using Google Scholar and from the University of Alberta library.

### 2) Examine the Meaning of the Theory

The key concepts of Peplau's theory were identified and defined theoretically, operationally, and descriptively. These concepts included: person (the individual/patient receiving care), nurse, environment, health, and the three sequential phases of the nurse-patient relationship (orientation, working, resolution).

### 3) Analyze the Logical Adequacy of the Theory

The logical structure of Peplau's theory was reviewed to confirm that the relationships between concepts were logically sound. Assessment as to whether or not the theory's structure allowed for logical predictions about nurse-patient interactions was undertaken.

### 4) Determine the Usefulness of the Theory

The practical application of Peplau's theory to clinical nursing practice was evaluated in context with literature on nurses enhancing older adults’ health literacy, which we retrieved from online databases and assessed in the context of Peplau's theory. PRISMA-S guidelines for literature searches (Rethlefsen et al., 2021) were followed. On September 22, 2024, three databases were searched: MEDLINE, CINAHL, and PsycINFO. These databases were selected due to their relevance to health sciences and wide indexing of nursing literature. No date limits were used in the search strategy. Table 1 presents the MEDLINE search strategy. All search strategies are provided in Supplemental File 1. A PRISMA 2020 flow diagram (Page et al., 2021) in Figure 1 depicts the screening process undertaken in Covidence.

**Figure 1.**
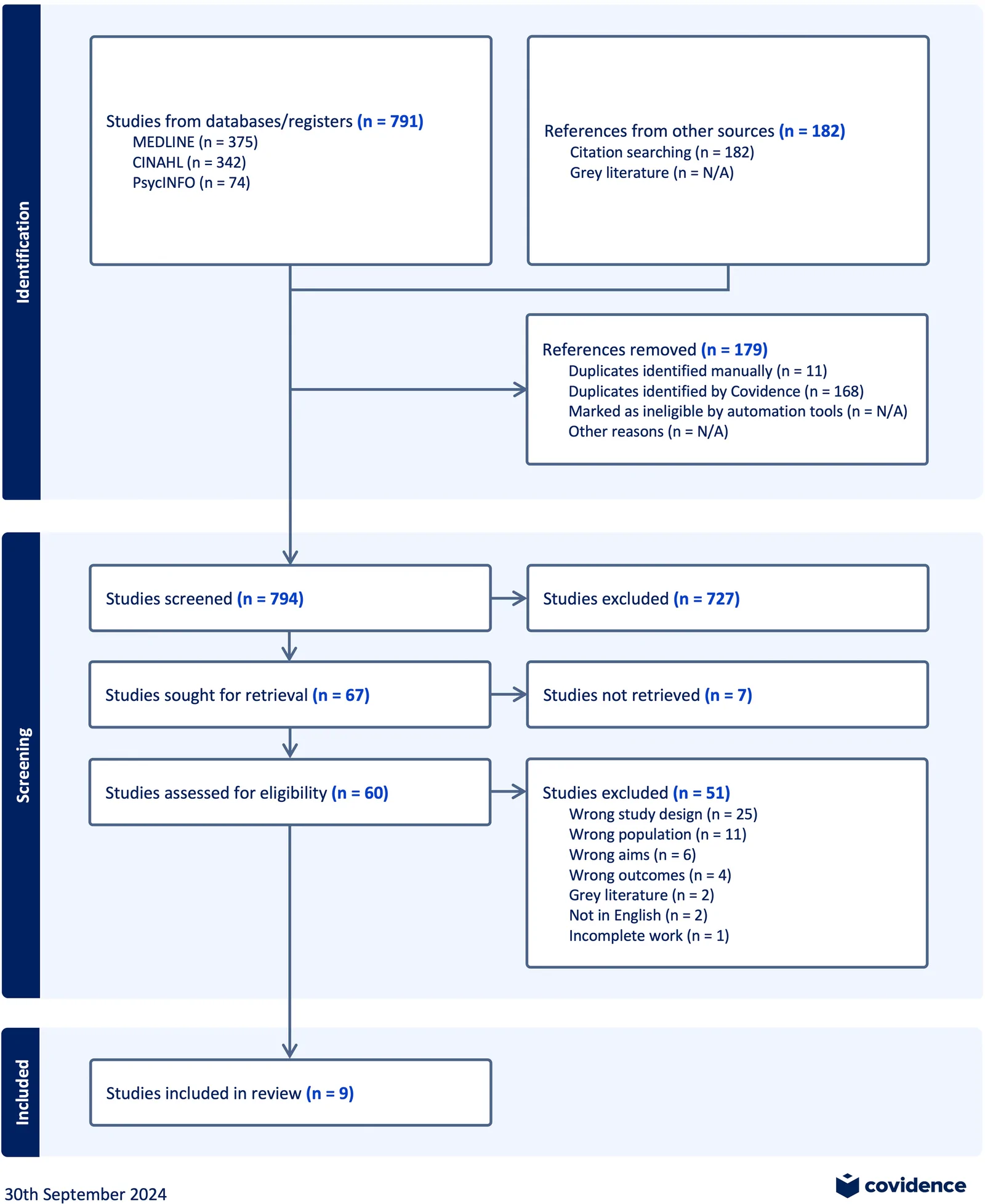
PRISMA 2020 flow diagram, generated in Covidence.

**Table 1.** MEDLINE (OVID Interface) Search Strategy, Conducted on September 22, 2024

#	Query	Results from Sep 22, 2024
1	Health Literacy/ or health literacy.mp.	20,126
2	exp Geriatrics/ or exp Aged/ or Health Services for the Aged/ or Senior Centers/ or (elders or elderly or geriatric* or gerontolog* or “old age” or “senior citizen*” or (seniors not “high school”) or ((older or mature) adj3 (adult* or person* or people or patient or patients or man or men or woman or women)) or centenarian* or nonagenarian* or octogenarian* or septuagenarian* or sexagenarian* or dottering or decrepit or tottering or overaged or “oldest old” or supercentenarian*).mp.	3,802,232
3	Nursing/ or nurs*.mp.	842,987
4	1 and 2 and 3	375

Inclusion criteria were qualitative or mixed methods original research; published in the English language; the population was nurses caring with older adults (sample mean age of >60 years or narratively identified as “older adults” within the text of the manuscript); the concept was the enhancement of health literacy; and the context was within the interpersonal relationship between the nurse and the older adult patient. Quantitative research did not meet the aims of this analysis and was excluded.

### 5) Define the Degree of Generalizability

The theory's generalizability and transferability were assessed and extrapolated within the included literature by exploring how existing literature described enhancing health literacy through nurse-patient relationships. We chose the three-step thematic synthesis method (Thomas & Harden, 2008) as a structured approach to analyze the included studies. This involved qualitative coding and creating descriptive and analytical themes to generate insights related to Peplau's Interpersonal Relations Theory that were not explicitly present in the included studies. The thematic synthesis was conducted by the first author and subsequently reviewed and confirmed by the second author.

### 6) Determine the Testability of the Theory

The testability of Peplau's theory was assessed by reviewing its ability to generate hypotheses related to health literacy enhancement within the nurse-patient relationship. To address this step, we provide implications for future research in our Discussion section.

## Findings

### Theory Origins

Peplau's Interpersonal Relations Theory was developed by Hildegard E. Peplau in 1952, and republished in 1988 and 1991 (Peplau, 1991). Peplau is renowned as a pioneering figure in the field of psychiatric nursing (Haber, 2000; Pearson, 2008) and is known to have significantly influenced nursing practice, education, and the professionalization of nursing, particularly through the development of her theory (Haber, 2000). Her work in the mid-20^th^ century brought new perspectives and approaches to nursing, moving away from the more custodial care practices of the time (Sills, 2009). It uses a straightforward process that mirrors natural human interactions and deductively builds on the psychodynamic theories of Harry Stack Sullivan and Sigmund Freud (Haber, 2000; Peplau, 1982; Thompson, 1986). Peplau emphasized the importance of the nurse-patient relationship as a therapeutic modality (Pearson, 2008). Her theory has been foundational in both the art and science of nursing practice, highlighting the therapeutic potential of effective communication and interactions between nurses and patients (Gastmans, 1998; Pearson, 2008). Her inductive approach to theorizing was based on keen observations from her clinical work, which helped to professionalize nursing and establish it as a distinct scientific discipline (Gastmans, 1998; Sills, 2009).

Reviewing seminal work, it is described that Peplau's theory was deductively constructed. The theory incorporates elements from both Harry Stack Sullivan's interpersonal theory (Sullivan, 2013) and Sigmund Freud's psychoanalytic theory to understand how conscious and unconscious thoughts influence behavior, particularly in the context of nursing practice and therapeutic relationships (Forchuk, 1993; Haber, 2000; Thompson, 1986). Regarding interpersonal theory, Sullivan theorized that personality develops through interpersonal relationships and that human behavior is shaped by interactions with others (Sullivan, 2013). Sullivan emphasized the role of anxiety in interpersonal dynamics, suggesting that anxiety is transferred or experienced through interactions, especially in significant relationships (Sullivan, 2013). Peplau extended this idea by framing the nurse-patient interaction as a core therapeutic tool (Peplau, 1991; Simpson & Peplau, 1991; Thompson, 1986). She argued that the nurse helps the patient address anxiety and develop healthier coping mechanisms by fostering trust, empathy, and understanding in their interactions (Thompson, 1986). Freud's psychoanalytic theory introduced the concept that unconscious thoughts, feelings, and experiences influence behavior (Holt, 1989). Peplau acknowledged this and recognized that patients’ reactions and behaviors in interpersonal settings could stem from unresolved unconscious conflicts (Peplau, 1991). Inspired by Freud's focus on transference and countertransference (Holt, 1989), Peplau stressed the importance of the nurse's self-awareness (Peplau, 1991). Nurses must recognize their own unconscious biases and emotional responses (countertransference) to maintain a professional and therapeutic relationship (Adams, 2017).

Ultimately, Peplau's theory is particularly valued for its focus on human issues in nursing, advocating for the importance of understanding patients’ unique experiences and perceptions (Deane & Fain, 2015; Hagerty et al., 2017). While initially developed in the context of psychiatric nursing, the theory has since been applied across various nursing settings, highlighting the importance of empathy, active listening, and mutual respect in fostering trust and achieving positive patient outcomes (Deane & Fain, 2015; Forchuk, 1993; Simpson & Peplau, 1991). This approach helps nurses holistically assess patients’ psychological, emotional, and spiritual needs, leading to more comprehensive and patient-centered care (Forchuk, 1993; Reynolds, 1997).

### Meaning and Logical Adequacy

#### Meaning

Peplau's Interpersonal Relations Theory is a middle-range nursing theory that focuses on the nurse-patient relationship as the foundation for effective nursing practice. It highlights the therapeutic process that occurs when nurses engage with patients in a structured and dynamic interpersonal interaction (Peplau, 1991, 1992). Concepts that are central to Peplau's theory are defined herein: person, nurse, environment, health, and the three sequential phases of the nurse-patient relationship.

**Person.** In Peplau's theory, the person (often referred to as the patient) is a developing individual striving to reduce anxiety caused by unmet needs. The person is central to the interaction, with growth and healing being shared goals. The theory encourages nurses to treat each person as unique and emphasizes personal dignity (Peplau, 1991, 1992, 1997).

**Nurse.** The nurse in Peplau's theory is defined as a professional who engages in a therapeutic relationship with the patient (Peplau, 1991). While many healthcare professionals engage in therapeutic relationships, the role of the nurse includes various phases and responsibilities, such as educator, resource person, counsellor, and leader. Through a dynamic interaction, the nurse helps the patient progress through the stages of health and healing (Peplau, 1991). Nurses are seen as facilitators of patient growth, not just caregivers who deliver treatment (Barker, 1998; Nyström, 2007).

**Environment.** Although Peplau's theory does not explicitly define “environment” in the same manner as some other nursing models, it implies that the environment includes all factors affecting the person's ability to engage in interpersonal relations, including social, cultural, and psychological aspects. The core difference in defining environment within Peplau's theory is that it focuses primarily on the nurse-patient relationship and the interpersonal processes that occur within it (Peplau, 1991). The therapeutic environment is created through the nurse-patient relationship, where communication, empathy, and understanding foster trust (Barker, 1998; Senn, 2012).

**Health.** Peplau defines health as, not just the absence of illness, but a dynamic and evolving state where individuals can achieve personal growth (Peplau, 1991), which is relevant to health literacy. The therapeutic nurse-patient relationship plays a critical role in enhancing the patient's health by helping them address and manage their health concerns (Barker, 1998; Nyström, 2007).

**Phases of the Nurse-Patient Relationship.** Lastly, Peplau's theory is divided into three distinct phases (Peplau, 1991):

1. *Orientation phase:* This is where the nurse and patient meet, and the patient recognizes their need for help. The nurse's role is to gather essential information and start building a relationship based on respect and trust. During this initial contact, both nurse and patient begin to shed or test any “preconceptions,” clarifying each other's role and the scope of the interaction (Forchuk, 2024; Peplau, 1991). The nurse explains confidentiality measures, potential time frames for meetings, and the importance of open communication (Peplau, 1991). These steps encourage the patient to see the nurse as capable, consistent, and trustworthy, setting the stage for meaningful engagement (Jojan & Carroll, 2024). This phase sets the foundation for the patient to view the nurse as someone capable of helping them through their health journey (Hagerty et al., 2017; Senn, 2012). However, the orientation phase is not always a smooth process (Forchuk, 1992; Peplau, 1991). If a patient does not recognize their information needs or need for help, this can become a risk to the development of a nurse-patient relationship (Forchuk, 1992), thus inhibiting the enhancement of health literacy. Viewing the patient holistically and understanding their worldview is key to developing a therapeutic relationship (Frey et al., 2013), especially in the orientation phase (Forchuk, 1992).
2. *Working phase:* This phase involves two subphases – identification and exploitation. Here, the nurse actively engages in problem-solving and patient education, assuming various roles such as caregiver, educator, or counselor depending on the patient's needs. In the identification subphase, patients move beyond surface issues and begin to share deeper concerns, increasingly recognizing the nurse as a dependable resource and collaborator (Forchuk, 2024; Peplau, 1991). In the exploitation subphase, the patient ‘makes full use’ of the nurse's expertise, such as practicing new behaviors, gathering feedback, and building real-world coping skills. During this critical work, the nurse continually adapts interventions (teacher, coach, or advocate) to match the patient's changing needs, fostering confidence and independence (Jojan & Carroll, 2024). The patient begins to identify with the nurse, seeing them as a trustworthy resource, and together, they work toward achieving health goals (Senn, 2012).
3. *Termination phase:* In the final phase, the relationship is concluded as the patient's immediate health needs are met, and they are prepared for discharge or independence. At this point, nurse and patient jointly review progress made and discuss how newly developed skills or knowledge can be maintained outside formal care. This reflective process often includes reassurance about community resources, preventive measures, and strategies to avoid setbacks (Peplau, 1991). The termination phase also validates the patient's autonomy, affirming their capacity for continued health management (Jojan & Carroll, 2024). The success of this phase depends on the nurse's ability to help the patient transition toward self-reliance (Barker, 1998; Senn, 2012).

#### Logical Adequacy

Peplau's theory realizes logical adequacy as it is rooted in established psychological and interpersonal theories from the time of its development. It emphasizes mutual interaction, focusing on the patient's active role in the relationship, which aligns with modern healthcare's goals of patient-centered care. Furthermore, the theory's focus on phases that evolve intuitively during therapeutic encounters makes it versatile across different care settings, such as psychiatric, emergency, and rural nursing, which rely heavily on effective communication (Senn, 2012). The theory encourages nurses to not only observe but also meaningfully interact with patients where there is a focus on patient-centeredness, and the needs, preferences, and values of the patient are prioritized, thus fostering positive outcomes by addressing both psychological and physiological needs (Deane & Fain, 2015; Hagerty et al., 2017).

### Usefulness and Generalizability

To determine the usefulness and generalizability of Peplau's Interpersonal Relations Theory, we conducted a database search. Nine studies were found to meet inclusion criteria, which explored the health literacy of older adults in the context of interpersonal relations with nurses. Although none of the included studies explicitly mentioned Peplau's theory, they did explore the concept of nursing's interpersonal relationship building for enhancing older adults’ health literacy. The characteristics of these studies are presented in Table 2.

**Table 2.** Study Characteristics

Author (Year)	Place of Study	Study Design	Data Collection	Study Aims	Main Themes	Implications for Practice and Interpersonal Communication
Brooks et al. (2020)	United Kingdom	Qualitative	Focus groups	Explore nursing and allied HCPs’ views about identifying and responding to older adults’ health literacy needs.	-Identifying older adults’ health literacy levels -Current practices for and barriers to responding to variations in older adults’ health literacy levels -Views about using health literacy screening and universal precautions	-Nurses and HCPs need to standardize their practice to health literacy by integrating strategies into routine practice. -Nurses and HCPs should use clear and accessible tailored communication, build trust, assess understanding, and involve patients’ social networks.
Cook et al. (2018)	New Zealand	Qualitative	Interviews	Understand women's perspectives about the trajectory from first bodily changes to diagnosis of endometrial cancer.	-Long-standing assumptions and beliefs -Communicating with health services and practitioners -Threshold between nothing and something -Health literacy	-There may be structural and communication barriers for women navigating healthcare systems -It is vital that nurses take the time to both listen to women and provide them with resources to enhance their health literacy.
Doekhie et al. (2020)	Netherlands	Mixed methods	Questionnaire and interviews	Explore how social context relates to how the patient perceives their role in the decision-making process.	-Subjective norms: underlying factors in the patient-informal caregiver/nurse relationship -Social support: networks of multiple support circles -Patient's role in the decision-making process: implicit and explicit patient role expectations	-Patients, informal caregivers, and nurses should be encouraged to discuss their mutual role expectations. -Nurses should take a tailored approach to patient/caregiver education.
Ferreira et al. (2022)	Portugal	Qualitative	Written narrative responses	Understand how nursing students’ experiences in a clinical teaching context contribute to the development of their competencies in the promotion of health and prevention of disease in older adults.	-Strategies to promote health and prevent disease in older adults -Health improvements from the implementation of the strategies -The impact of participation in these strategies to promote health and prevent disease on the formative process of nursing students	Strategies for health promotion and disease prevention adapted to and focused on the needs of older adults should be implemented into teaching in undergraduate nursing education.
Forsgärde et al. (2023)	Sweden	Qualitative	Interviews	Describe the support for all involved in acute situations when a community health nurse is called, as experienced by older adults, their significant others, and nurses involved.	-Knowledge of temporality as support in decisions -Confidence as support in past and present relations -Decision support in the present situation -Dependency on mutual interpersonal support	-Establishing a structured collaboration between nurses and the wider healthcare team promotes informed care decisions for the older adult by leveraging each profession's respective expertise and organizational advantages. -Enhance continuity of relationships in home healthcare settings to facilitate the use of temporality as a form of support.
Goeman et al. (2016)	Australia	Mixed methods	Questionnaire and interviews	Improve a service's approach to diabetes education so that clients are better supported to self-manage their condition.	-Benefits experienced during the use of diabetes education checklist -Benefits and barriers experienced during the use of teach-back -Benefits and barriers experienced during the use of the online library of resources -Benefits and barriers experienced during the use of the learning styles tool	A structured method that supports healthcare services to co-design interventions that respond to the health literacy needs of their clients can lead to evidence-informed, sustainable practice changes that support clients to better understand effective diabetes self-management.
Kim and Oh (2020)	Korea	Qualitative	Interviews and email exchange	Understand nurses’ perspectives on the education of older patients and their health literacy.	-Attitudes that are hard to change -Physical and cognitive functional barriers to understanding teaching materials -Family caregivers: surrogate vs. gatekeeper -Major contexts that moderate elderly people's health literacy -Strategies to enhance teaching effectiveness and health literacy	Systemic assistance and interventions specialized for older patients and their HCPs need to be developed and tested to improve clinical practice and patient health literacy.
Marca-Frances et al. (2020)	Spain	Qualitative	Focus groups and interviews	Identify from the perspectives of patients which topics matter most, who should be communicating them, and how should they be provided?	-Hospitalization procedure -Health literacy around patient's condition -Information content -Satisfaction -Professional-patient relationship -Patient proactivity	Ensure effective communication with and comprehension in patients.
Saenyabutr et al. (2024)	Thailand	Qualitative	Focus groups and interviews	Understand the community people's experience of access, understanding, evaluation, and application of health information for self-care of hypertensive older adults and explore the community's perspective on promoting community health literacy.	Themes to promote health literacy: -Usual healthcare activities -Community action -Nurses at primary care unit should be involved in playing a key role in health literacy promotion	Nurses and community committees should collaborate to ensure older adults in the community have better health literacy, which will lead to better self-care of hypertension and improved health outcomes.

### Thematic Synthesis

#### Descriptive Themes

From the initial coding, four descriptive themes were named. These included (1) building trust through communication; (2) health literacy as a joint effort; (3) the role of social support; and (4) overcoming health literacy barriers.

##### Building Trust through Communication

The relationship between nurse and patient is grounded in effective communication (Doekhie et al., 2020; Goeman et al., 2016). Trust is a key element, particularly when addressing complex health information. Nurses must adopt patient-centered communication styles, which allow them to engage older adults and build a trusting therapeutic relationship (Doekhie et al., 2020; Goeman et al., 2016). Furthermore, familiarity with patients’ preferences and history enhances the effectiveness of care delivery and receptiveness to health teaching (Doekhie et al., 2020). Brooks et al. (2020) highlight that clear, accessible communication is critical for building trust between nurses and older adults, especially when addressing complex health literacy needs. Nurses’ abilities to use subtle cues to assess health literacy and their approach to communication play a key role in developing a trusting therapeutic relationship (Brooks et al., 2020). Forsgärde et al. (2023) discuss how relational continuity and mutual interpersonal support build trust in acute healthcare situations. When nurses know their patients well and communicate effectively, it fosters confidence and trust, which are crucial in health decision-making (Forsgärde et al., 2023).

##### Health Literacy as a Joint Effort

The included studies highlight that improving health literacy is a shared responsibility between nurses and older adults. Nurses are not only providers of care but also key facilitators of patient understanding. For instance, Goeman et al. (2016) and Ferreira et al. (2022) illustrate that when nurses engage older adults in health education using tailored strategies, comprehension and management of chronic conditions improve. Goeman et al. (2016) discuss how improving health literacy is a collaborative process between nurses and older adults. In their study, nurses worked with older adults to co-design interventions tailored to their individual health literacy needs, demonstrating that both parties must actively participate to improve health literacy, particularly in the management of chronic conditions such as diabetes. Marca-Frances et al. (2020) highlight how nurse-patient collaboration is essential for enhancing health literacy during hospitalization. During hospitalization, older adults depend on nurses and doctors to explain their health conditions (Marca-Frances et al., 2020). Effective health literacy requires two-way communication and joint effort between older adults and their healthcare professionals (Marca-Frances et al., 2020).

##### The Role of Social Support

Doekhie et al. (2020) and Saenyabutr et al. (2024) iterate that social support plays a pivotal role in enhancing health literacy. The involvement of family and friend caregivers and community support systems is critical for older adults who rely on external help to understand and follow medical instructions. This network of family and friend caregivers can bridge the gap between healthcare professionals and older adults, ensuring consistent information reinforcement (Cook et al., 2018; Doekhie et al., 2020). Doekhie et al. (2020) explore how social support, particularly from nurses and family and friend caregivers, significantly influences older adults’ decision-making processes. Older adults who perceive strong social support are more likely to take an active or shared role in their health decisions, while those who feel expected to leave decisions to healthcare professionals often adopt a passive role (Doekhie et al., 2020). Saenyabutr et al. (2024) emphasize the role of community involvement in promoting health literacy, especially in the context of older adults with chronic conditions like hypertension. Social support from family, community health volunteers, and local nurses plays a crucial role in ensuring older adults access, understand, and apply health information effectively (Saenyabutr et al., 2024). Goeman et al. (2016) discuss the importance of social support for home care patients. Their study highlights how family and friend caregivers and nurses can help patients better understand and manage their health through tailored interventions and consistent support (Goeman et al., 2016).

##### Overcoming Health Literacy Barriers

Health literacy barriers are multifaceted, including physical limitations, cognitive decline, and systemic issues within healthcare organizations (Kim & Oh, 2020). Nurses must adapt their educational strategies to the specific needs of each patient, ensuring comprehension despite these barriers (Forsgärde et al., 2023; Kim & Oh, 2020). Kim and Oh (2020) discuss how physical and cognitive impairments, such as memory decline and reduced cognitive function in older adults, present barriers to understanding health information. Nurses must address these barriers by simplifying communication and using interactive techniques like teach-back methods (Kim & Oh, 2020). Forsgärde et al. (2023) highlight how nurses can address barriers in acute situations, especially for older patients who may face challenges like fear, lack of control, and cognitive difficulties. The interpersonal support from nurses, who know the patient's medical history and communication needs, can mitigate these barriers.

**Analytical Themes.** The final step of thematic synthesis involves synthesizing the descriptive themes into broader analytical themes that reflect the role of the interpersonal relationship between nurses and patients in enhancing health literacy. These three analytical themes are: (1) the interpersonal relationship as a medium for health literacy enhancement; (2) empathy and relational continuity as essential elements; and (3) nurses as key facilitators of learning.

***The Interpersonal Relationship as a Medium for Health Literacy*.** Across the included articles, it is evident that the nurse-patient relationship is a key mediator in the promotion of health literacy. Nurses who build strong, trusting relationships with their older adult patients create an environment conducive to learning. When nurses take time to understand the older adult's communication needs and barriers, they can significantly enhance the patient's ability to manage their own health. Brooks et al. (2020) emphasize that nurses’ interpersonal relationships with older adults are essential for addressing health literacy challenges. Through effective communication and relationship-building, nurses can assess patients’ literacy needs and provide tailored health information, creating a supportive environment for improving health outcomes. Goeman et al. (2016) illustrate that the relationship between home care nurses and older adults plays a significant role in enhancing health literacy, especially in cases of chronic disease management. When nurses engage in sustained and supportive interactions, older adults are better equipped to understand and apply health information (Goeman et al., 2016). Forsgärde et al. (2023) highlight the importance of relational continuity, where long-term relationships between nurses and older adults help facilitate trust and improve communication. This continuity enables nurses to provide personalized health literacy support, improving decision-making in acute healthcare situations. Kim and Oh (2020) discuss the role of nurses’ interpersonal communication skills in improving health literacy among older adults. By building strong relationships with patients and understanding their cognitive and physical challenges, nurses can more effectively convey complex health information. These articles demonstrate that the interpersonal relationship between nurses and patients is key to facilitating enhanced health literacy, as it fosters trust, personalized care, and ongoing communication that empower patients to manage their health more effectively.

***Empathy and Relational Continuity as Essential Elements*.** Studies such as Forsgärde et al. (2023) and Goeman et al. (2016) show that empathy and continuity of care are fundamental in fostering health literacy. Older adults are more likely to engage in self-care and adhere to treatment plans when they feel understood and supported by their healthcare professionals (Forsgärde et al., 2023; Goeman et al., 2016). Forsgärde et al. (2023) emphasize relational continuity, which allows nurses to develop a deep understanding of the patient's medical history, preferences, and communication needs. This continuity fosters empathy and better decision-making, particularly in acute situations (Forsgärde et al., 2023). Goeman et al. (2016) discuss how empathy and sustained, supportive interactions between home care nurses and patients lead to improved health literacy. When nurses show empathy and take the time to understand their patients’ needs, patients are more likely to engage in self-management and better comprehend their health information. Lastly, Kim and Oh (2020) highlight that nurses create an environment of trust by consistently demonstrating empathy and investing in building strong personal relationships with their older adult patients. This relational continuity ensures that nurses can effectively convey health information, overcoming cognitive and physical barriers. The findings from these studies indicate that empathy and maintaining long-term relationships with patients are key elements in enhancing health literacy, as they lead to trust, a better understanding of patient needs, and improved communication.

***Nurses as Key Facilitators of Learning*.** Nurses are uniquely positioned to act as educators for their patients because of their initial point of contact and high-level engagement throughout the care continuum. By leveraging teach-back methods, tailored interventions, and supportive communication techniques, they can guide patients through the complexities of their health conditions (Ferreira et al., 2022; Marca-Frances et al., 2020). Ferreira et al. (2022) and Marca-Frances et al. (2020) both demonstrate how structured educational interventions led by nurses can empower patients to manage their health more effectively. Ferreira et al. (2022) highlight that nursing students, through their clinical teaching experiences, develop strategies to promote health literacy and disease prevention. This emphasizes that nurses, both in training and practice, play a key role in educating patients over time, facilitating learning by adapting their teaching to the needs of older adult patients. Marca-Frances et al. (2020) discuss how nurses, through constant interaction with patients, serve as primary educators. Their role in explaining health conditions and procedures during hospitalization promotes continuous patient learning, ensuring that patients are empowered to manage their health beyond the immediate hospital setting. These articles demonstrate that nurses are not only care providers but also essential educators who support older adults in gaining and applying health knowledge, and facilitating health literacy and self-care skills.

## Discussion

### Testability of the Theory

Peplau's theory is inherently testable because it focuses on interactional processes, communication, and role development between nurses and patients. These can be empirically observed and measured in practice. While Peplau's theory has been directly tested within the literature in different contexts (Fathidokht et al., 2023; Forchuk, 1992, 1994; McNaughton, 2005; Thompson, 1986), this theory analysis demonstrates the *indirect* testability of Interpersonal Relations Theory, applied in the context of enhancing the health literacy of older adults.

### Implications for Future Research

The descriptive themes outlined in the synthesis provide clear pathways for hypothesis generation. For example, “*building trust through communication*” might lead to a hypothesis such as “Older adults who experience consistent, patient-centered communication from nurses will have higher levels of health literacy compared to those who do not receive personalized communication.” This hypothesis can be tested by measuring trust-building communication strategies, such as frequency, clarity, and patient-specific adjustments, and evaluating their effect on health literacy outcomes in older adults. In the case of “*health literacy as a joint effort*,” a nursing scientist might hypothesize “Collaborative nurse-patient health education will result in improved health literacy and better self-management of chronic conditions in older adults.” The joint effort described suggests an interactive and dynamic process where both nurse and patient co-create health strategies. This collaborative process can be observed and tested by assessing health literacy improvements before and after tailored health interventions in the nurse-patient interaction.

The analytical themes also support hypothesis generation. Considering the “*interpersonal relationship as a medium for health literacy*,” Peplau's theory asserts that the therapeutic relationship is fundamental to all nursing actions. The thematic synthesis confirms this, showing that strong nurse-patient relationships improve health literacy. Testing this can involve examining different qualities of the nurse-patient relationship (e.g., empathy) and their direct impact on literacy outcomes. Another testable aspect is relational continuity. For example, long-term nurse-patient relationships could be hypothesized to foster deeper health literacy improvements compared to short-term or transient relationships. This aligns with Peplau's emphasis on the evolving phases of interaction in the interpersonal process. Lastly, considering “*nurses as key facilitators of learning*,” Peplau's description of the nurse's role as a teacher can be tested by assessing how nurse-led health education strategies, such as teach-back, improve patient outcomes. This directly relates to the hypothesis that structured, consistent nurse-patient education enhances health literacy.

### Implications for Clinical Practice

Practice implications arise from this theory analysis. Peplau's theory underscores the importance of developing strong therapeutic relationships between nurses and older adults. These relationships are key to building trust, which in turn enhances effective communication and health literacy. Building on our descriptive theme of “*building trust through communication,”* nurses can leverage the interpersonal relationship as a medium for health literacy by actively engaging older adults in dialogue, assessing their unique comprehension needs, and creating a supportive environment for shared decision-making (Brooks et al., 2020; Forsgärde et al., 2023; Goeman et al., 2016). Nurses should invest in developing these interpersonal relationships to create an environment where older adults feel comfortable and empowered to engage in their health decisions. An exploration of how to do this effectively would be beneficial to an individual nurse's growth in clinical practice. Furthermore, nurses need to adapt their communication strategies based on the older adults’ individual needs, health literacy levels, and cognitive capacities. This includes using teach-back methods, simplifying health information, and adjusting communication styles to ensure understanding. Such approaches recognize that “*health literacy is a joint effort”* and underscore “*nurses as key facilitators of learning,”* which are themes from our analysis that highlight the collaborative nature of nurse-patient interactions in promoting older adults’ understanding of complex health information (Ferreira et al., 2022; Marca-Frances et al., 2020).

Considering the reported finding that health literacy is a shared responsibility, nurses should involve older adults in the learning process, making health education a collaborative effort. This empowers older adults to participate actively in managing their health. In alignment with our analytical theme of “*empathy and relational continuity as essential elements,”* nurses can demonstrate consistent support and understanding of older adults’ experiences to foster lasting trust (Forsgärde et al., 2023; Goeman et al., 2016). By maintaining ongoing relationships and being attentive to patients’ evolving needs, nurses reduce health-related anxiety and enhance receptiveness to education, thereby improving overall health literacy outcomes. Lastly, nurses should integrate family and friend caregivers into the health literacy education process. The inclusion of social support systems can enhance understanding and reinforce health information for older adults, who often rely on their support networks for assistance in healthcare management. Emphasizing the “*role of social support”* not only aligns with Peplau's relational focus but also addresses potential barriers that older adults may encounter (Kim & Oh, 2020; Saenyabutr et al., 2024).

### Implications for Holistic Nursing

Holistic nursing emphasizes the integration of body, mind, spirit, and the environment to promote comprehensive patient well-being (Frisch & Rabinowitsch, 2019). Peplau's Interpersonal Relations Theory aligns with the principles of holistic nursing, providing a framework that prioritizes patient-centered care and the therapeutic relationship as fundamental to improving health outcomes. The theory's focus on the nurse-patient relationship as a therapeutic process supports a holistic nursing goal of addressing not just physical health needs but also emotional, psychological, and social dimensions of care. Nurses can foster a meaningful connection that empowers patients to take an active role in their health by understanding the patient as a whole person while considering their experiences, preferences, and values. This aligns with a holistic focus on empowering individuals through tailored communication, mutual respect, and shared decision-making (Muscat et al., 2021).

Peplau's phases of interaction (orientation, working, and termination) mirror the processes used in holistic nursing to build trust, engage collaboratively, and ensure continuity of care. In the orientation phase, relationships between nurses and patients often include inherent power inequities due to differences in knowledge and control over care processes (Baptista et al., 2018). However, holistic nursing approaches can help mitigate these imbalances by fostering a more equitable, patient-centered dynamic. Holistic nursing practice helps to alleviate feelings of powerlessness and foster a collaborative care environment (Kubsch & Wichowski, 1997). Furthermore, a holistic approach encourages nurses to view patients as whole persons rather than just their medical conditions. This involves respecting patients’ autonomy, engaging them in care decisions, and addressing emotional, social, and spiritual needs (Frisch & Rabinowitsch, 2019; Muscat et al., 2021). In relation to health literacy, approaching nursing from a holistic perspective can significantly improve patient knowledge and engagement (Menichetti et al., 2019). As well, during the working phase, nurses’ roles as educators and counselors address the emotional and cognitive needs of older adults, reinforcing the principles of holistic nursing. By using interpersonal strategies, such as active listening and empathy, nurses can enhance patient’s health literacy (Carollo, 2015).

Peplau's theory's adaptability allows for opportunity to be applied across diverse cultural and individual contexts, resonating with holistic nursing's recognition of the uniqueness of each patient (Frisch & Rabinowitsch, 2019). By incorporating Peplau's framework, holistic nursing practice is strengthened through an evidence-based approach that nurtures the physical, emotional, social, and intellectual health of patients, especially in cases of older adults navigating complex healthcare systems. This integration demonstrates how holistic nursing and Peplau's theory complement each other, providing a robust basis for improving health literacy and fostering a supportive, empowering healthcare experience for older adults.

### New Knowledge Contribution

This theory analysis offers contributions that advance nursing knowledge within Peplau's well-established theoretical framework. To our knowledge, this work is the first analysis to situate the health literacy of older adults within the phases of Peplau's nurse-patient relationship. We demonstrate the theory's enduring adaptability to lay conceptual groundwork for measuring relational health literacy practices in future studies. Our thematic synthesis operationalizes Peplau's concepts into examples of how her theory can be applied in practice. Nurses in clinical settings can adopt the phases of Peplau's theory (orientation, working, termination) to strengthen their interpersonal relations with older adults as they work to enhance health literacy within this population. Lastly, our review of the literature exposes a gap with only nine studies linking nurse-patient relationships to older adult health literacy. Therefore, an agenda for future research can emerge from this work.

### Strengths and Limitations

Strengths of this theory analysis include that the steps outlined by Walker and Avant (2019) were followed, while also adhering to PRISMA-S guidelines for literature searches (Rethlefsen et al., 2021) to broaden the examination of the theory in clinical nursing practice. Limitations include the fact that a single screener conducted the literature review, and that included literature did not explicitly state that Peplau's Interpersonal Relations Theory was a framework in the studies. Therefore, researcher bias may exist for including the study and interpreting it as such. While only nine studies were included in this theory analysis, a strength of this manuscript is highlighting the gap in the literature surrounding interpersonal relations as being structural for nurses enhancing the health literacy of older adults.

## Conclusion

In general, Peplau's theory remains relevant today due to its structured yet adaptable framework, which allows for dynamic and holistic nurse-patient relationships across various clinical settings. Its logical flow through its three phases provides a clear, replicable method for achieving therapeutic goals in nursing. This theory analysis sought to apply Peplau's theory to the enhancement of older adults’ health literacy. By examining the theory in this context, we are able to better understand the dynamic process that exists between nurses and older adult patients. By incorporating the holistic nursing focus on physical, emotional, social, and spiritual dimensions of a patient, Peplau's theory is suited to meet the context of present-day nursing. Furthermore, this analysis affirmed that leveraging the phases of the nurse-patient relationship fosters not only patient empowerment and active participation in care but also facilitates improved health literacy outcomes. Thus, the continued integration of Peplau's Interpersonal Relations Theory in nursing practice supports a patient-centered, holistic approach that is essential for addressing the evolving needs of older adults in complex healthcare systems.

## Supplemental Material

sj-docx-1-jhn-10.1177_08980101251345027 - Supplemental material for Applying Peplau's Interpersonal Relations Theory to Enhance Older Adults’ Health Literacy Through Nursing Care: A Theory Analysis Supplemental material, sj-docx-1-jhn-10.1177_08980101251345027 for Applying Peplau's Interpersonal Relations Theory to Enhance Older Adults’ Health Literacy Through Nursing Care: A Theory Analysis by Steven Hall and Kathleen Shearer in Journal of Holistic Nursing

## Data Availability

Included records within this theory analysis are available from the original publishers.
