# Supplementary material for: Applying Peplau's Interpersonal Relations Theory to Enhance Older Adults’ Health Literacy Through Nursing Care: A Theory Analysis
Source: J Holist Nurs. 2025 Jun 17;44(4):402–18. doi: 10.1177/08980101251345027 (PMC13714210; doi:10.1177/08980101251345027)
Supplement: Supplementary file 1 [file sj-docx-1-jhn-10.1177_08980101251345027.docx]

**Applying Peplau’s Interpersonal Relations Theory to Enhance Older Adults’ Health Literacy through Nursing Care: A Theory Analysis**

Supplementary File 1: Search Strategies

**Ovid MEDLINE(R) Epub Ahead of Print and In-Process, In-Data-Review & Other Non-Indexed Citations and Daily <September 19, 2024>**

1	Health Literacy/ or health literacy.mp.	20126

2	exp Geriatrics/ or exp Aged/ or Health Services for the Aged/ or Senior Centers/ or (elders or elderly or geriatric* or gerontolog* or "old age" or "senior citizen*" or (seniors not "high school") or ((older or mature) adj3 (adult* or person* or people or patient or patients or man or men or woman or women)) or centenarian* or nonagenarian* or octogenarian* or septuagenarian* or sexagenarian* or dottering or decrepit or tottering or overaged or "oldest old" or supercentenarian*).mp.	3802232

3	Nursing/ or nurs*.mp.	842987

4	1 and 2 and 3	375

**APA PsycInfo <1806 to September 2024 Week 3>**

1	Health Literacy/ or health literacy.mp.	7957

2	exp Geriatrics/ or exp Aged/ or Health Services for the Aged/ or Senior Centers/ or (elders or elderly or geriatric* or gerontolog* or "old age" or "senior citizen*" or (seniors not "high school") or ((older or mature) adj3 (adult* or person* or people or patient or patients or man or men or woman or women)) or centenarian* or nonagenarian* or octogenarian* or septuagenarian* or sexagenarian* or dottering or decrepit or tottering or overaged or "oldest old" or supercentenarian*).mp.	205450

3	Nursing/ or nurs*.mp.	138990

4	1 and 2 and 3	74

**CINAHL Plus with Full Text**

| **#** | **Query** | **Results** |
| --- | --- | --- |
| S1 | (MH Health Literacy) or ("health literacy") | 12,871 |
| S2 | (MH "Geriatrics") or (MH "Aged, Hospitalized") or (MH "Aged+") or (MH "Senior Centers") or (MH "Gerontologic Care") or (MH "Geriatricians") OR (MH "Gerontologic Nursing+") OR (MH "Health Services for the Aged") or ("aging in place" or elders or elderly or geriatric* or gerontolog* or gerodontic* or "old age*" or (seniors not "high school") or "senior citizen*" or (older N3 (patient* or adult* or person* or people or man or men or woman or women)) or centenarian* or nonagenarian* or octogenarian* or septuagenarian* or sexagenarian* or dottering or decrepit or tottering or overaged or "oldest old") | 1,085,872 |
| S3 | (MH Nursing) or (nurs*) | 1,000,412 |
| S4 | ((MH Nursing) or (nurs*)) AND (S1 AND S2 AND S3) | 342 |
